# Alzheimer’s Disease: Analyzing the Missing Heritability

**DOI:** 10.1371/journal.pone.0079771

**Published:** 2013-11-07

**Authors:** Perry G. Ridge, Shubhabrata Mukherjee, Paul K. Crane, John S. K. Kauwe, Alzheimer’s Disease Genetics Consortium

**Affiliations:** 1 Department of Biology, Brigham Young University, Provo, Utah, United States of America; 2 ARUP Institute for Clinical and Experimental Pathology, Salt Lake City, Utah, United States of America; 3 Department of Medicine, University of Washington, Seattle, Washington, United States of America; McGill University Department of Neurology and Neurosurgery, Canada

## Abstract

Alzheimer’s disease (AD) is a complex disorder influenced by environmental and genetic factors. Recent work has identified 11 AD markers in 10 loci. We used Genome-wide Complex Trait Analysis to analyze >2 million SNPs for 10,922 individuals from the Alzheimer’s Disease Genetics Consortium to assess the phenotypic variance explained first by known late-onset AD loci, and then by all SNPs in the Alzheimer’s Disease Genetics Consortium dataset. In all, 33% of total phenotypic variance is explained by all common SNPs. *APOE* alone explained 6% and other known markers 2%, meaning more than 25% of phenotypic variance remains unexplained by known markers, but is tagged by common SNPs included on genotyping arrays or imputed with HapMap genotypes. Novel AD markers that explain large amounts of phenotypic variance are likely to be rare and unidentifiable using genome-wide association studies. Based on our findings and the current direction of human genetics research, we suggest specific study designs for future studies to identify the remaining heritability of Alzheimer’s disease.

## Introduction

Alzheimer’s disease (AD) is the most common form of dementia. Worldwide estimates of prevalence vary, with estimates of 24 to 35 million people affected [1-3]. Combined with an aging population, prevalence is expected to increase to 1 in 85 people by 2050 [2]. 

AD is a heterogeneous disease caused by a combination of environmental and genetic factors. The most important risk factor for Alzheimer’s disease is age [1,4]. Environmental risk factors include hypertension, estrogen supplements [5], smoking [6,7], stroke, heart disease, depression, arthritis, and diabetes [8]. In addition, certain lifestyle choices appear to decrease the risk of AD: exercise [9], intellectual stimulation [10], and maintaining a Mediterranean diet (including fish)[11,12].

The genetics of AD are complex. Several genes are known to harbor either causative or risk variants for AD. There are two primary types of AD as defined by age. The first is early-onset, or familial AD, and the second type is late-onset AD (LOAD), sometimes termed sporadic AD. Three genes, *APP* [13], *PSEN1* [14], and *PSEN2* [15] are known to harbor many highly penetrant, autosomal dominantly-inherited variants, which lead to early-onset AD but account for only a small fraction of total AD cases.

LOAD accounts for 99% of AD cases and is caused by a more complex underlying genetic architecture. Genome-wide association studies (GWAS) have identified 10 different loci associated with AD (Table 1). Recent applications of next-generation sequencing (NGS) have suggested rare variants play important role and have large effects in the etiology of AD [16-18]. Identifying additional variants will provide information that is integral to the development, evaluation and application of effective therapeutic strategies for AD. Lee et al. [19] used 3,333 cases and 3,924 controls, including 2,699 population-based controls to estimate that common genetic variants account for 24% of variance in AD. They also estimated the contribution of APOE using several proxy SNPs, with varying degrees of LD, with the APOE ε4 allele to estimate the APOE effect at approximately 4%. Here we evaluate the variance in AD status explained by common SNPs and along with all recently identified AD genes, including direct genotyping of the APOE ε2 and ε4 alleles, in 5,708 AD cases and 5,214 clinically ascertained controls. We also suggest strategies for identifying the remaining AD genes.

**Table 1:** Late-onset Alzheimer’s disease associated genes/variants.

**Variant**	**Gene**	**Abbreviation**	**Odds Ratio**
rs429358	Apolipoprotein E (ε4 allele)[58]	APOE	3.685
rs7412	Apolipoprotein E (ε2 allele)[59]	APOE	0.621
rs744373	Bridging Integrator 1[32]	BIN1	1.166
rs11136000	Clusterin[33,60]	CLU	0.879
rs3764650	ATP-binding cassette, sub-family A (ABC1), member 7[31]	ABCA7	1.229
rs3818361	Complement component (3b/4b) receptor 1 (Knops blood group)[60]	CR1	1.174
rs3851179	Phosphatidylinositol binding clathrin assembly protein[33]	PICALM	0.879
rs610932	Membrane-spanning 4-domains, subfamily A, member 6A[31]	MS4A6A	0.904
rs3865444	CD33 molecule[20,31]	CD33	0.893
rs670139	Membrane-spanning 4-domains, subfamily A, member 4E[31]	MS4A4E	1.079
rs9296559	CD2-associated protein[20,31]	CD2AP	1.117

The dbSNP identification number, gene name, gene abbreviation, and odds ratio for each of the top variants from the Alzgene.org meta-analyses. The SNP in CD2AP, rs9349407, is not present in this sample, so we used rs9296559 instead as a proxy. These two SNPs are close together (1,108 base pairs apart) and in very high LD (r^2^=1).

## Methods

### Dataset

We used the Alzheimer’s Disease Genetics Consortium (ADGC) dataset described in Naj et al. [20] for our analyses. Samples were genotyped using Affymetrix and Illumina SNP chips. Quality control of the imputed data was performed as described by Naj et al. 2011 [20]. Briefly, markers with a minor allele frequency of less than 1% and deviation from HWE where P<10^-6^ were removed. To have a common set of SNPs across all samples, imputation to HapMap phase 2 (release 22)[21] was performed using MaCH [22] and strand ambiguous SNPs were removed, resulting in a rectangular dataset with 2,042,114 SNPs. Only SNPs imputed with *R* ^2^ ≥ 0.50 were included in the dataset. We added an additional two SNPs, rs7412 and rs429358, corresponding to *APOE* ε2 and ε4, respectively. 

We used a compiled dataset of directly genotyped SNPs common to all 15 studies to assess cryptic relatedness and calculate principal components to account for population-specific variations in allele distribution. We excluded strand ambiguous SNPs, resulting in a rectangular dataset with 21,880 directly observed (not imputed) SNPs in common across all the studies. We filtered SNPs with pairwise LD (*r* ^2^) < 0.20, resulting in a dataset with 17,054 SNPs. We used both PLINK [23] and KING-ROBUST [24] for relatedness analysis. KING-ROBUST provided unbiased kinship coefficient estimates for related individuals in our dataset. We excluded up to 3^rd^ degree relatives (kinship >= 0.0442) for a final dataset containing 19,692 individuals.

Of the 19,692 individuals in the original dataset we analyzed a subset of 10,922 individuals who had complete data for the 11 markers listed in Table 1, AD case-control status, age, sex, and 10 principal components from the population stratification analysis (missingness rates for each of the covariates and case-control status are reported in Table S1). Basic demographic information for the 10,922 individuals in the subset of the dataset used in this study is presented in Table 2. We collected chromosome length and number of genes per chromosome from the Vega database [25].

**Table 2:** Demographic information for individuals in the analysis dataset.

	**Count**	**Cases / Controls**	**Age (Cases / Controls)**
**Male**	4489	2378/2111	74.8 (73.6 / 76.1)
**Female**	6433	3330/3103	75.3 (74.9 / 75.7)
**Total**	10922	5708/5214	75.1 (74.3 / 75.9)

We report total individuals, sex, case-control status, and average age for the 10,922 individuals analyzed in this report.

### Genetic Analyses

We used Genome-wide Complex Trait Analysis (GCTA)[26], a tool that implements the methods described in Yang et al. [27], Lee et al. [28], and Yang et al. [29] to estimate the phenotypic variance explained by known AD genes and tagged by SNPs on the SNP arrays. Briefly, GCTA uses a mixed linear model and treats the effects of SNPs as random effects, effectively testing all the SNPs together for effect (in contrast to GWAS, which considers each SNP individually). We used age, sex, and 10 principal components as covariates. For the analyses in which we examined unexplained phenotypic variance, we also controlled for the 11 known AD markers (Table 1). The 11 known AD markers are the AlzGene.org top hits and are the markers with replicable evidence for association with AD. Each of these markers is present in our dataset except rs9349407 in CD2AP. As proxy we used rs9296559, which is in very high LD with rs9349407 (r^2^=1). We specified a population prevalence of LOAD at 0.13 [30].

### Ethics Statement

All study procedures were approved by the Institutional Review Boards of Brigham Young University and the University of Washington.

## Results

**Figure 1:**
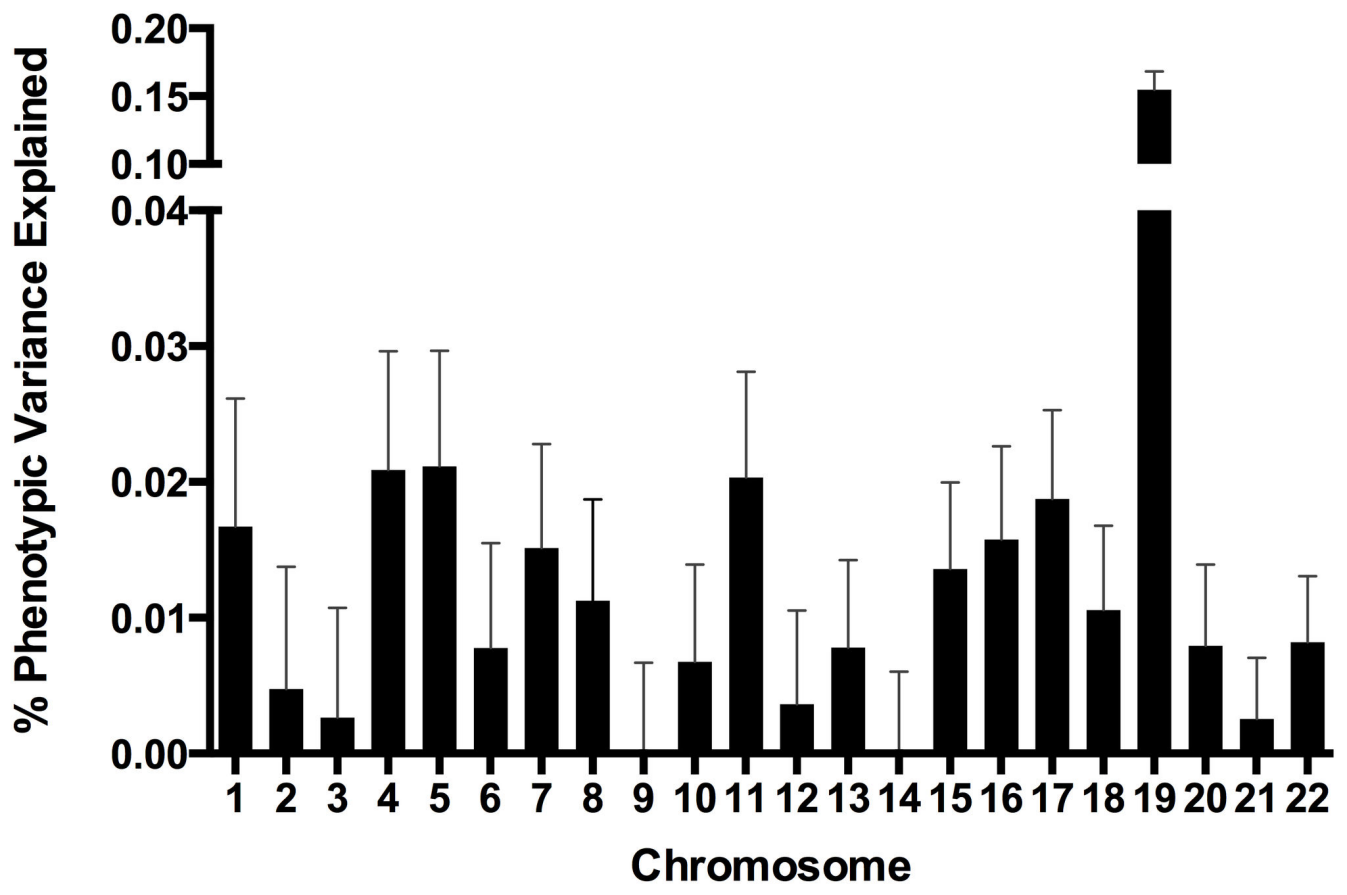
Unexplained Alzheimer’s disease variance, by chromosome. In this figure we show phenotypic variance, by chromosome, explained by all SNPs. Error bars correspond to standard error.

We estimated the variance in AD case-control status focusing first on the 11 known AD markers (Table 1). Together these markers account for 7.8% (standard error 0.03) of the phenotypic variance (Table 3). Next, we estimated the explained phenotypic variance for each chromosome (Figure 1). Chromosome 19 accounts for the highest proportion of phenotypic variance. 

**Table 3:** Summary of genetic and phenotypic variance measurements.

**SNP Set**	**Genetic Variance (Standard Error)**	**% Phenotypic Variance (Standard Error)**
All SNPs	0.071 (0.0072)	33.12% (0.033)
APOE ε2/ε4	0.020 (0.0066)	5.92% (0.033)
All known AD markers (Table 1)	0.025 (0.0066)	7.78% (0.033)
All SNPs excluding known AD markers	0.046 (0.0060)	25.34% (0.033)

In this table we summarize our results, showing genetic and percent phenotypic variance for four different subsets of SNPs: all SNPs in the ADGC dataset, the two APOE alleles (ε2 and ε4), all known AD markers (as listed in Table 1), and all SNPs excluding those in Table 1.

**Figure 2:**
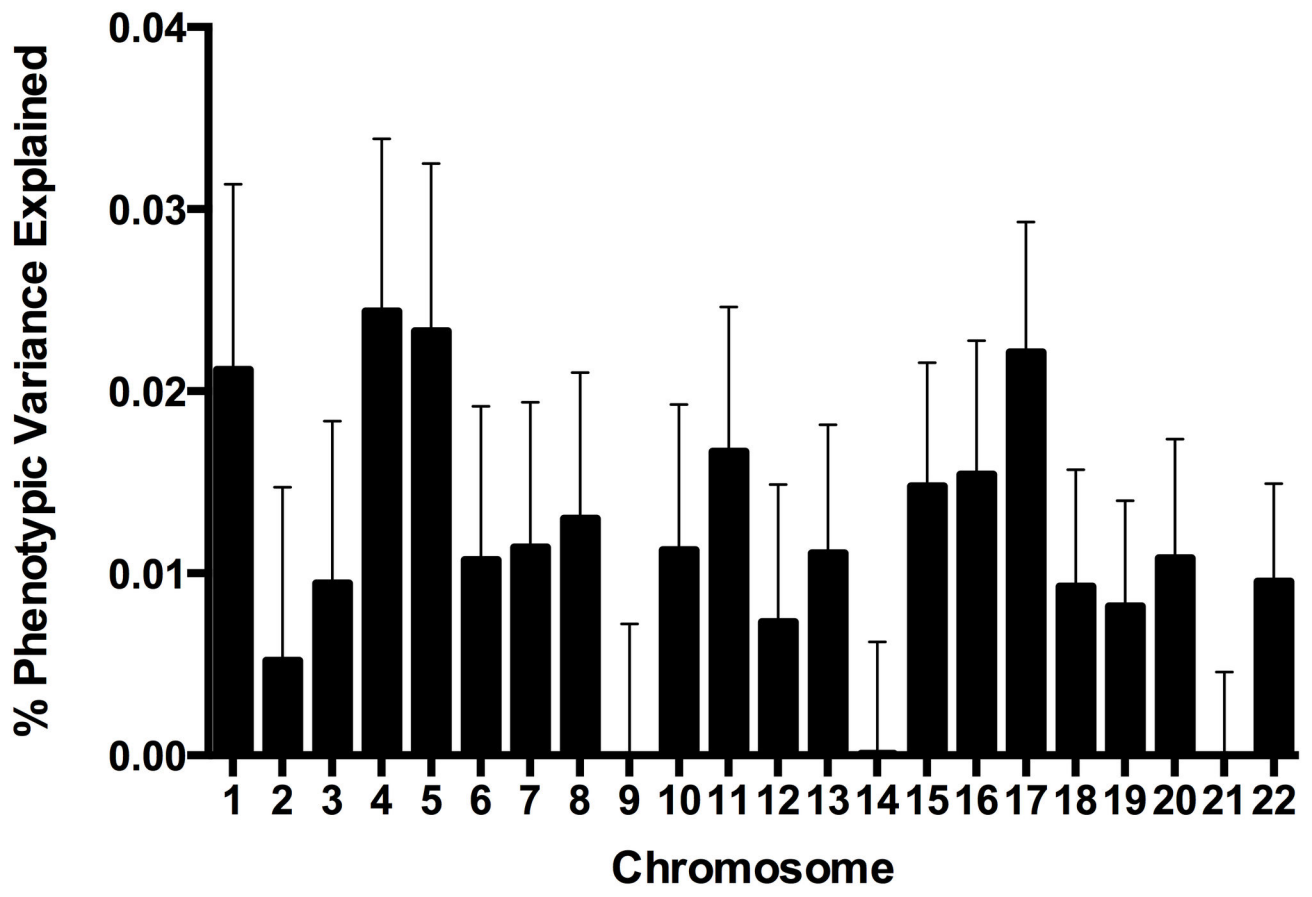
Unexplained Alzheimer’s disease variance, by chromosome, excluding known Alzheimer’s disease markers. This figure is the same as Figure 1 except we have removed variance explained by known Alzheimer’s disease markers. Error bars correspond to standard error.

In all, the 2,042,116 SNPs in the HapMap imputed ADGC dataset explain 33.1% of phenotypic variance (genetic variance of 0.0711, standard error 0.0072). The APOE ε2 and ε4 alleles account for 5.9% (standard error 0.03) of the phenotypic variance (Table 3). The other 9 known high frequency SNPs identified in GWAS explain an additional 1.9% (standard error 0.03)(Table 3). After controlling for these 11 markers, an additional 25.3% of the total phenotypic variance (genetic variance of 0.046, standard error 0.006) is explained with as-yet unidentified variants (Table 3). The remaining phenotypic variance explained by each chromosome after controlling for the 11 known markers is shown in Figure 2. SNPs on chromosomes 1, 4, 5 and 17 account for the largest percentage of remaining unexplained phenotypic variance compared to other chromosomes, each accounting for more than 2% (Figure 2). Chromosomes 9, 14, and 21 account for the least (<0.0001% each); however, there is unexplained phenotypic variance on all the autosomes. There is no relationship between explained variance and chromosome length (p-value = 0.8), or number of genes per chromosome (p-value = 0.7). 

## Discussion

**Figure 3:**
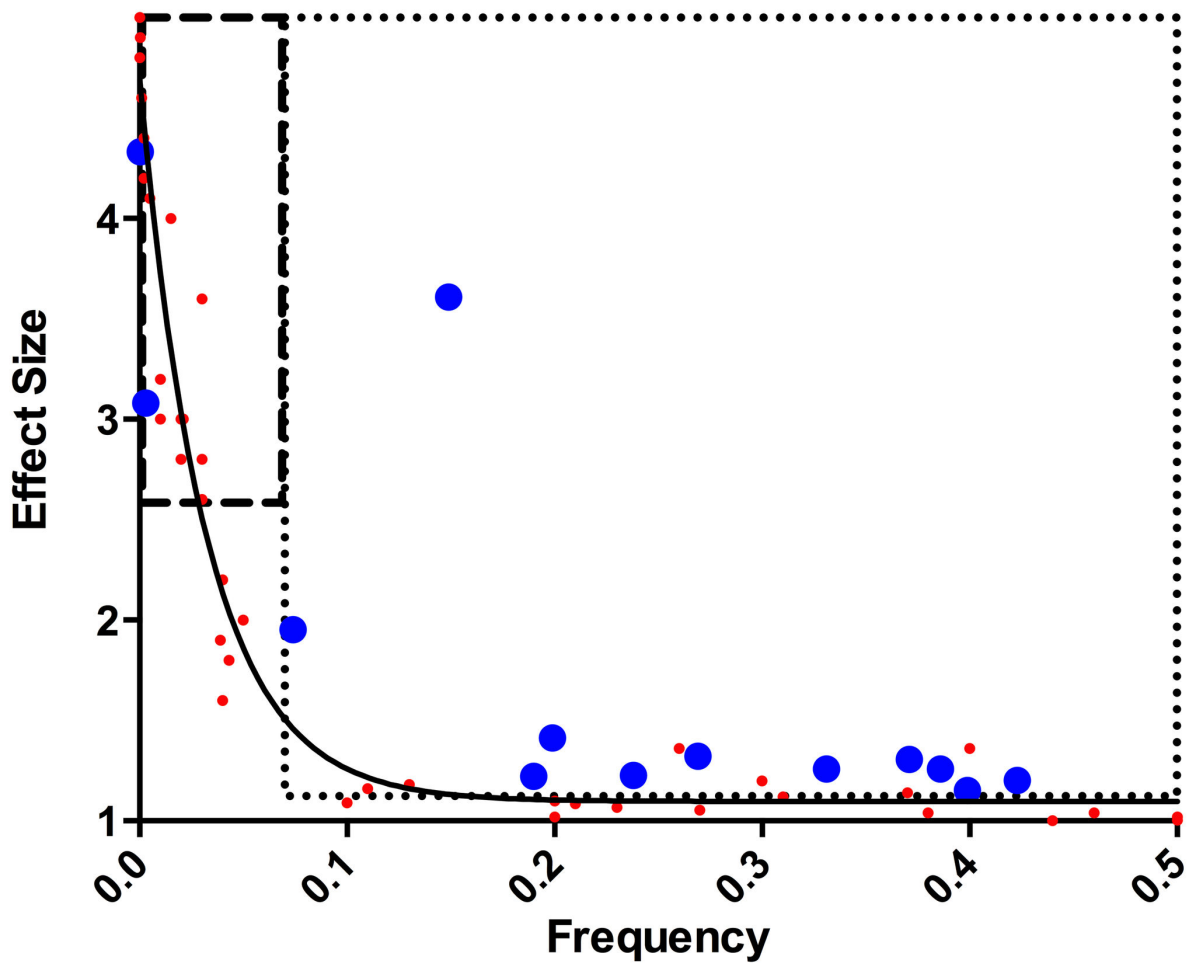
Variant search space. Real and hypothetical variants are graphed by effect size (y-axis) and population frequency (x-axis). Known Alzheimer’s disease SNPs are blue circles and hypothetical SNPs are red circles. The large box on the right outlined with dots, is the GWAS search space and the smaller box on the left, outlined with dashes, is the next-generation sequencing search space. Known Alzheimer’s disease SNPs are those found in Table 1 as well as APP and TREM2, which are both labeled on the graph.

A clear understanding of the genetic architecture of Alzheimer’s disease provides the foundation of information needed to cure this terrible disease. While many large GWAS for AD have been performed and several replicable loci have been identified (as referenced in Table 1), relatively little phenotypic variance is explained by these variants. Our estimates of phenotype variance explained by common genetic variants and by the APOE locus are higher than those of Lee et al. [19]. We estimated total phenotypic variance explained by common SNPs to be ~33%. In contrast Lee et al. [19] estimated ~24%. In our study we used genotyped and HapMap imputed SNPs, whereas Lee et al. [19] used only directly genotyped SNPs. Inclusion of imputed SNPs improved heritability estimates and suggests that imputed SNPs should be included in such studies. In addition to using imputed variants, our dataset was larger and our controls were clinically ascertained. Differences in the estimates for APOE (~6% in this study compared to ~4% in Lee et al. [19]) could be due to these same characteristics as well as the direct genotyping of the APOE ε2 and ε4 alleles in our samples as opposed to the use of proxy markers. Regardless, both studies provide evidence that a considerable amount of variance in AD is explained by yet unidentified genetic variation. Together these results show that there is clearly much work to be done if we are to solve the genetic architecture of AD. GWAS with sample sizes performed to date are able to identify common variants with moderate to small effect sizes. Results of GWAS in AD and other conditions suggest there may be a large number of such variants and that additional variants of this type can be identified by increasing sample sizes. However, additional loci detected with the GWAS strategy will likely have effects either similar to or smaller than SNPs already identified. The range of SNPs identifiable by current GWAS [20,31-36] is marked on Figure 3 by the large box bordered by dots (the GWAS search space), with recent GWAS hits inside the labeled oval. The GWAS being conducted by the International Genomics of Alzheimer’s Project represents a substantial increase in sample size and will undoubtedly identify additional common loci with small effects on AD risk. Nevertheless, it is unlikely that many common variants of even modest effect size remain to be identified. 

There are still many AD variants that remain to be identified, however, and these variants exist on every autosome (Figure 2). Variants with large effects are almost certainly present in very low frequencies or they would have been identified in GWAS. While such variants are unlikely to be detected using traditional GWAS due to limitations of r^2^ based “tagging” for alleles with different frequencies [37] the current analysis allows for high D’ values between common alleles and rare variants of large effect to contribute to the explained variance. These rare variants of large effect appear in the smaller box bordered with dashes in Figure 3. To date, identified alleles of this type have clear functional effects and large effect sizes compared to associated alleles from GWAS. Detecting rare variants of large effect requires different experimental designs than GWAS such as sequencing causal loci. Exome chip array studies target known variation in coding regions, even those of very low frequency; this may prove a promising and economical approach. However, accurately genotyping variants of less than 1-2% using these arrays is quite challenging, and for variants that are present below these frequencies other approaches are required. 

Two seemingly contradictory hypotheses exist about the architecture of complex disease: the common disease/common variant hypothesis and the multiple rare variant hypothesis. In the first, many common variants of small effect size collectively explain disease risk, while in the second, rare variants, some with large effect and high penetrance, explain disease risk. However, as suggested by Singleton et al. [38] these two hypotheses are not mutually exclusive and the genetics of complex diseases are likely a hybrid of the two. Singleton et al. [38] suggests that both common and rare variants that increase or decrease disease risk are likely to be found in the same loci and coined the phrase “pleomorphic risk loci”. To date, AD genetics research has largely focused on common variants that influence disease risk, likely due to technological and financial constraints. However, the advent of next generation sequencing (NGS) and falling costs of this technology have made it possible to expand AD research to include searching for rare variants. Recently, this technology was used to identify a functional variant that protects against Alzheimer’s disease in the amyloid precursor protein (APP)[17]. Additionally, two groups recently used NGS to identify additional, likely functional, variants associated with AD in the triggering receptor expressed on myeloid 2 (*TREM2*) gene [16,18]. The *TREM2* variant is present in about 1% of the general population and has a high odds ratio (2.9 to 5.1 depending on the dataset). Likewise, the *APP* variant is extremely rare (frequency of 0.00038), but confers a large protective effect on carriers. Larger scale applications of this technology and careful study design are likely to identify additional variants and further explain the remaining phenotypic variance in AD.

Family-based studies are also an effective application of NGS. These studies require carefully ascertained families and accurate pedigree data and can be used to identify high effect, low frequency variants (located in the box with longer dashes in Figure 3). Family-based studies are especially powerful because large effect, low frequency disease-causing (or disease-modifying) sequence features, some of which may be unique to a single family, are likely to segregate, at least partially, with disease status. These approaches have not yet been extensively applied in AD research. Nevertheless, family-based studies utilizing large-scale genome or exome sequencing have recently been used to identify disease-causing variants in several Mendelian [39-41] and complex disorders [42,43].

It is also possible that gene-gene interactions account for much of the unexplained variance in AD status [44]. These interactions are widespread and common [45,46] and approaches to understand the effects of epistatic interactions exist and continue to mature [44,47]. Several interesting candidate interactions have been identified and Ebbert et al. 2013 (accepted) recently demonstrated that allowing interactions improves the diagnostic utility of the known AD markers. Unfortunately, the complexity of this problem and the extremely large samples sizes required to perform agnostic screens for gene-gene interactions make it very difficult to conduct effective screens for these effects. 

AD is a highly complex disease with substantial genetic and environmental components. Our results suggest that genetic variance accounts for ~30% of phenotypic variance, but over 75% of this phenotypic variance remains unexplained by currently identified AD genes. Future AD genetics research must leverage larger samples and novel technologies such as NGS to identify rare, high penetrant variation and gene-gene interactions that are likely to explain the remaining genetic and phenotypic variance in AD. 

Genetic research in AD has followed roughly the same model as the study of other complex diseases; largely focusing on the identification of common variants of modest effect using association studies. Scientist in many disease fields have successfully identified numerous associated variants (this is a small representative sample [48-57]). The transition from a focus on common variants to a focus on the identification of low frequency variants is now underway. These rare, functionally relevant markers are often more easily characterized than common variants of small effect. This will lead to strong and testable hypotheses for the development of therapeutics, thus accelerating the progress toward effective prevention and treatment.

## Supporting Information

Table S1 **Missingness rates for covariates and case-control status.** The Alzheimer’s Disease Genetics Consortium dataset consists of 19,692 total individuals. We removed any individuals missing any of the covariates (listed here) or case-control status (included in this table).
(DOCX)
