# Supplementary material for: Alzheimer’s Disease: Analyzing the Missing Heritability
Source: PLoS One. 2013 Nov 7;8(11):e79771. doi: 10.1371/journal.pone.0079771 (PMC3820606; doi:10.1371/journal.pone.0079771)
Supplement: Table S1 — Missingness rates for covariates and case-control status. The Alzheimer’s Disease Genetics Consortium dataset consists of 19,692 total individuals. We removed any individuals missing any of the covariates (listed here) or case-control status (included in this table). (DOCX) [file pone.0079771.s001.docx]

**Supplementary Table 1. Missingness rates for covariants and case-control status**

| **Covariates/Case-control Status** | **# of individuals missing the given covariate (% missing)** |
| --- | --- |
| Age | 1491 (7.57%) |
| Sex | 0 (0%) |
| Case-control Status | 0 (0%) |
| APOE (rs429358/rs7412) | 579 (2.94%) |
| 10 Principal Components | 0 (0%) |
| BIN1 (rs744373) | 2872 (14.58%) |
| CLU (rs11136000) | 3009 (15.28%) |
| PICALM (rs3851179) | 211 (1.07%) |
| MS4A6A (rs610932) | 30 (0.15%) |
| CD33 (rs3865444) | 392 (1.99%) |
| MS4A4E (rs670139) | 192 (0.98%) |
| CD2AP (rs9296559) | 2374 (12.06%) |
| CR1 (rs3818361) | 54 (0.27%) |
| ABCA7 (rs3764650) | 2964 (15.05%) |

The Alzheimer’s Disease Genetics Consortium dataset consists of 19,692 total individuals. We removed any individuals missing any of the covariates (listed here) or case-control status (included in this table).
